# Coordinated Changes in Mutation and Growth Rates Induced by Genome Reduction

**DOI:** 10.1128/mBio.00676-17

**Published:** 2017-07-05

**Authors:** Issei Nishimura, Masaomi Kurokawa, Liu Liu, Bei-Wen Ying

**Affiliations:** Graduate School of Life and Environmental Sciences, University of Tsukuba, Ibaraki, Japan; Korea Advanced Institute of Science and Technology

**Keywords:** genome reduction, mutation rate, growth rate, genome size, experimental evolution

## Abstract

Genome size is determined during evolution, but it can also be altered by genetic engineering in laboratories. The systematic characterization of reduced genomes provides valuable insights into the cellular properties that are quantitatively described by the global parameters related to the dynamics of growth and mutation. In the present study, we analyzed a small collection of W3110 *Escherichia coli* derivatives containing either the wild-type genome or reduced genomes of various lengths to examine whether the mutation rate, a global parameter representing genomic plasticity, was affected by genome reduction. We found that the mutation rates of these cells increased with genome reduction. The correlation between genome length and mutation rate, which has been reported for the evolution of bacteria, was also identified, intriguingly, for genome reduction. Gene function enrichment analysis indicated that the deletion of many of the genes encoding membrane and transport proteins play a role in the mutation rate changes mediated by genome reduction. Furthermore, the increase in the mutation rate with genome reduction was highly associated with a decrease in the growth rate in a nutrition-dependent manner; thus, poorer media showed a larger change that was of higher significance. This negative correlation was strongly supported by experimental evidence that the serial transfer of the reduced genome improved the growth rate and reduced the mutation rate to a large extent. Taken together, the global parameters corresponding to the genome, growth, and mutation showed a coordinated relationship, which might be an essential working principle for balancing the cellular dynamics appropriate to the environment.

## IMPORTANCE

Genome reduction is a powerful approach for investigating the fundamental rules for living systems. Whether genetically disturbed genomes have any specific properties that are different from or similar to those of natively evolved genomes has been under investigation. In the present study, we found that *Escherichia coli* cells with reduced genomes showed accelerated nucleotide substitution errors (mutation rates), although these cells retained the normal DNA mismatch repair systems. Intriguingly, this finding of correlation between reduced genome size and a higher mutation rate was consistent with the reported evolution of mutation rates. Furthermore, the increased mutation rate was quantitatively associated with a decreased growth rate, indicating that the global parameters related to the genome, growth, and mutation, which represent the amount of genetic information, the efficiency of propagation, and the fidelity of replication, respectively, are dynamically coordinated.

## INTRODUCTION

The mutation rate is an essential global parameter, representing the plasticity and/or evolution of the genomic background. The mutation rate, reflecting the *in vivo* baseline of the DNA replication error rate, is different from species to species (1) but might be altered within the same population, from either low to high mutation rates (2, 3) or high to low mutation rates (4, 5), during a time scale of experimental evolution. Thus, the mutation rate is not only a force for adaptive evolution but is also itself able to evolve. In an evolutionary view on mutation rate, a correlation between the mutation rate and the genome size was intriguingly observed in eubacteria, archaea, and double-stranded DNA (dsDNA) viruses (6). If the evolution of mutation rates is coordinated with genome size, an intriguing question arises regarding whether genome reductions performed in the laboratory can be linked to mutation rates.

Genome reduction is a powerful approach (7) to explore essential working principles in living systems (8) and to determine basic genetic information (9, 10). The successful construction of an assortment of reduced genomes using *Escherichia coli* cells (11–13) has not only benefitted biotechnology in terms of protein syntheses (14, 15) and metabolic engineering (16) but also has led to significant progress in understanding the genome-wide and/or evolutionary properties of bacterial cells (17–20). The latest systematic surveys have reported a correlation between genome reduction and growth rate (19), a representative global parameter representing the activity of living cells. According to these novel findings, the following questions arose regarding whether and how genome reduction influences other fundamental properties of living cells, such as the mutation rate and the relationships among the global parameters.

To address these questions, we examined experimentally the mutation rates of the reduced genomes and observed a quantitative relationship between genome reduction and mutation rate in the present study. In addition, the evolvability of the mutation rate associated with the fitness change was demonstrated by a short-term experimental evolution with a reduced genome. Coordination between mutation and growth rates induced by genome reduction was clearly identified, which indicated a universal relationship among the three global parameters of genome size, mutation rate, and growth rate.

## RESULTS AND DISCUSSION

### Increased mutation rate induced by genome reduction.

Ten strains of varied genome lengths (see Fig. S1A in the supplemental material), comprising the wild-type genome (4.6 Mb) and nine reduced genomes (deletions of 89 to 982 kb) randomly selected from the W3110 reduced genome collection KHK (13) were analyzed. The mutation rates of these strains, grown in three different media, namely, LB, M63, and MAA (M63 supplemented with 20 amino acids) as the nutritional variation, were repeatedly measured.

10.1128/mBio.00676-17.1 FIG S1  Mutation rates and growth rates of the reduced genomes**.** (A) Genome length. The 10 W3110 *E. coli* strains with either the wild-type genome (no. 0) or the reduced genomes (nos. 3 to 28) are shown. (B) Mutation rates and growth rates. The 10 genomes are indicated with their collection numbers. Blue, orange, and red circles indicate results for M63, MAA, and LB media, respectively. Standard deviations are indicated. Download FIG S1, PDF file, 0.1 MB. Copyright © 2017 Nishimura et al. 2017 Nishimura et al. This content is distributed under the terms of the Creative Commons Attribution 4.0 International license.

**FIG 1:**
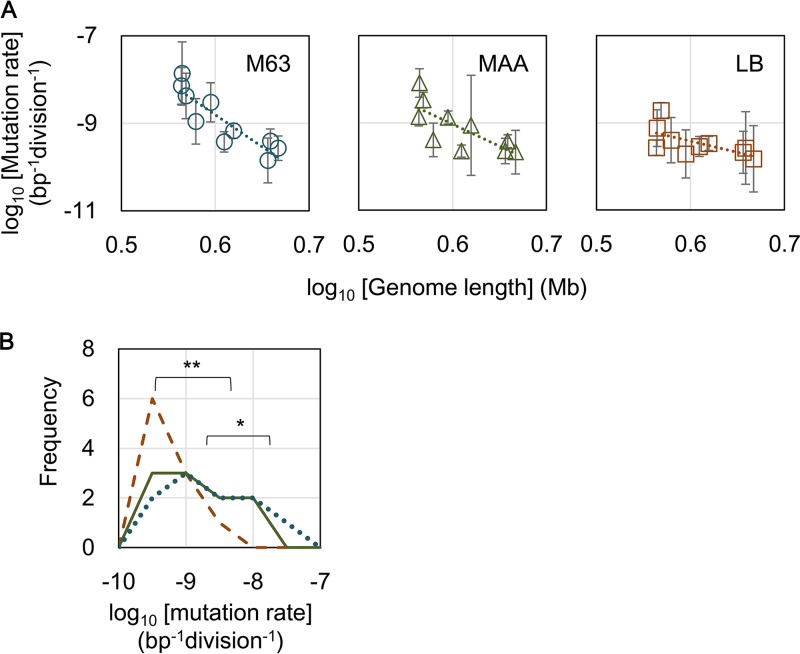
Correlations between genome reduction and mutation rate**.** (A) Genome length-correlated changes in mutation rates. The wild-type genome of strain W3110 (no. 0) and its reduced genomes (nos. 3, 4, 10, 11, 14, 19, 23, 27, and 28) are indicated. The results in the different growth media, LB, MAA, and M63, are shown. Spearman rank correlation coefficients of the mutation rates and the genome sizes were −0.891 (*P* = 0.001), −0.830 (*P* = 0.003), and −0.636 (*P* = 0.05) in M63, MAA, and LB, respectively. Standard deviations of the results from repeated measurements are shown. Dotted lines indicate the linear regression of the logarithmic genome sizes and the logarithmic mutation rates. The slopes of the mutation rate lines were as follows: −14.6 (*r*^*2*^ = 0.79), −10.1 (*r*^*2*^ = 0.57), and −5.1 (*r*^*2*^ = 0.41) for M63, MAA, and LB, respectively. (B) Direction of nutrition-dependent changes in the mutation rate. Distributions of the mutation rates in M63, MAA, and LB media are shown as dotted blue, solid green, and broken brown lines, respectively. Frequency on the *y* axis indicates the number of strains with the indicated mutation rate. Asterisks indicate significance (*, *P* < 0.05; **, *P* < 0.01).

Interestingly, an increase in the mutation rate with genome reduction was identified (Fig. 1A), although the genes participating in the DNA replication fidelity and mismatch repair systems remained in the genomes of these strains. Significant correlations between the mutation rates and genome reductions were identified in all three media (*P* < 0.05), suggesting that the genome reduction-mediated changes in the mutation rate were independent of nutrition. The magnitude of the genome reduction correlated with changes in the mutation rate on the order of nutritional levels; that is, larger magnitudes were observed in poorer media (Fig. 1A). We assumed that the genome reduction potentially stimulated the replication errors, reflecting the maladaptation of the deletion of redundant genetic information. Such stress might be compensated by nutritional richness, as the mutation rates of the cells grown in LB remained approximately at regular levels (Fig. 1A, right panel). This assumption was consistent with the fact that the distribution of the mutation rates of these 10 strains significantly shifted from high to low (*P* < 0.05) in response to the nutritional alterations from poor to rich (Fig. 1B), although the changes in the individual strains were somehow different (Fig. S1B).

### A common rule of genome size-correlated changes in the mutation rate.

The results interestingly suggested that the correlation of the genome length with changes in the mutation rate is a common rule, not only in the evolution of the mutation rate in prokaryotes (6) but also in the engineering of genome reductions with a defined *E. coli* strain. DNA content analysis indicated that the number of genes was a major factor influencing the size of the prokaryotic genome (21). Accordingly, genome size enlargement during the evolution of free-living bacteria generally increases the number of genes, and this is correlated with the number of regulators (22). The correlation between a decrease in the mutation rate and an increasing size of the genome was assumed to be beneficial for maintaining novel regulators or regulatory mechanisms that evolved to promote efficient growth. However, the genome reduction-mediated increase in the mutation rate was induced by the stress of the disappearance of the genomic sequences, although these sequences were somehow redundant for living. Despite these differentiated reasons, the correlations were universally detected, on a time scale either as long as evolution or as short as genetic manipulation. Simple regression, as applied for the study on evolution of the mutation rate (6), was performed to estimate the rate of the genome length-correlated changes in the mutation rate (Fig. 1A, dotted lines), according to the following formula: log_10_(*M*_*i*_) = *r*[log_10_(*G*_*i*_)] + *b.*

Here, *M*_*i*_ and *G*_*i*_ denote the mutation rate and the genome size (in megabases), respectively. The rates (*r*) of the genome size-correlated changes in the mutation rate were −5.1, −10.1, and −14.6 in LB, MAA, and M63 medium, respectively (Fig. 1A, slopes of the dotted lines). These rates (*r*) were similar to previously reported genome reduction-correlated changes in the growth rate (19). The results indicated that the magnitudes of the genome size-correlated changes in both the mutation rates and growth rates were dependent on nutritional conditions. In addition, these rates were much higher (approximately −1.1) than those estimated for evolution across eubacteria, archaea, and dsDNA viruses (6). Thus, the magnitude of the genome size-correlated changes in the mutation rate somehow reflected the time scale of the genomic changes, which was reasonable, as short-term genetic engineering brings more severe stress to the cells than does long-term evolution.

### Gene functions related to the increased mutation rate.

**FIG 2:**
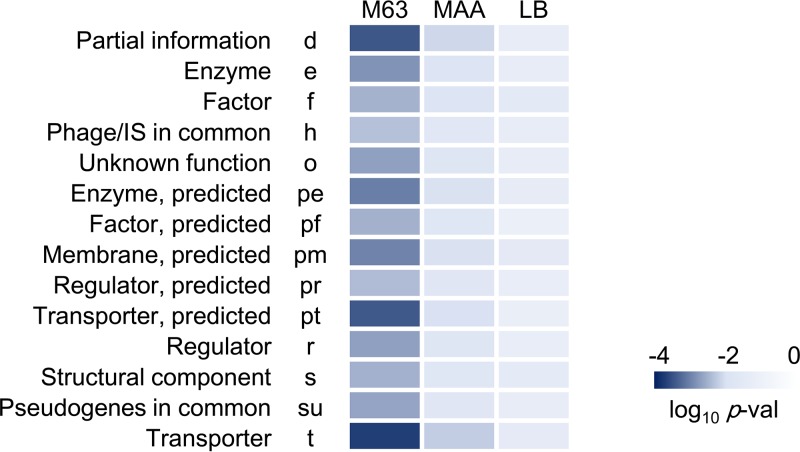
Correlation between the mutation rate and the number of deleted genes. The deleted genes were clustered according to the gene categories. Statistical significance levels for the correlation coefficients between the mutation rates and the numbers of deleted genes in each gene category are presented as a heat map on a logarithmic scale. The progression from dark to light blue represents significance levels from high to low. M63, MAA, and LB are the growth media. Gene categories are noted by both their full names and their corresponding abbreviations.

To identify whether any gene categories involved in the deleted genome sequences specifically contributed to the increase in mutation rate induced by genome reduction, gene function enrichment and correlation analyses were performed. The genes located within the deleted genomic regions were classified into 23 gene categories (23). The number of genes assigned to each category and the accumulated number of deleted genes in each reduced genome were counted, as previously described (19). The gene categories comprising more than 10 deleted genes in from the wild-type genome no. 28 (14 of the 23 gene categories) were subjected to a correlation analysis. The correlations between the increasing mutation rates found in bacteria grown in three different types of medium (Fig. 1A, left) and the increasing numbers of deleted genes in each gene category were evaluated (Table S1). Relatively high significant differences (*P* < 0.01) were detected in all 14 gene categories for bacteria cultured in M63 compared to those cultured in LB, which indicated that most genes contributed to the increased mutation rates observed under poor nutritional conditions (Fig. 2). In particular, the highest significance was identified in the gene categories for partial information (d), transporter (t), and predicted transporter (pt) proteins when cells were grown in M63 medium (*P* < 5e−4) and MAA medium (*P* < 0.01), suggesting that the disturbance in transport machineries and conserved proteins potentially triggered increased errors in DNA replication. Intriguingly, the gene categories d and t, together with that of predicted membrane (pm) also showed the highest significance in the correlation between the growth rate and the number of deleted genes (reported previously [19]). Taken together, the changes in the mutation rate induced by genome reduction appear to be coordinated with the changes in growth rate and largely occur due to deletion of genes that encode proteins involved in transport and membranes.

10.1128/mBio.00676-17.4 TABLE S1  Numbers of deleted genes in each gene category (The genes located within the deleted genomic regions were counted and classified into 23 gene categories, which are shown by their single-letter abbreviations; the number of genes assigned to each category is also indicated. In the table, the assigned number of reduced genomes, the accumulated number of deleted genes in strain genome nos. 3 to 28, and the total number of genes assigned to each gene category in the wild-type genome [genome no. 0] are represented in the columns listed as Strain No, Total del, and All genes, respectively. The gene categories shown in bold [14 out of 23] were subjected to the correlation analysis shown in Fig. 2 in the main text. This table was modified from the previous report [19].) Download TABLE S1, DOC file, 0.1 MB. Copyright © 2017 Nishimura et al. 2017 Nishimura et al. This content is distributed under the terms of the Creative Commons Attribution 4.0 International license.

### Correlation between mutation and growth rates in reduced genomes.

**FIG 3:**
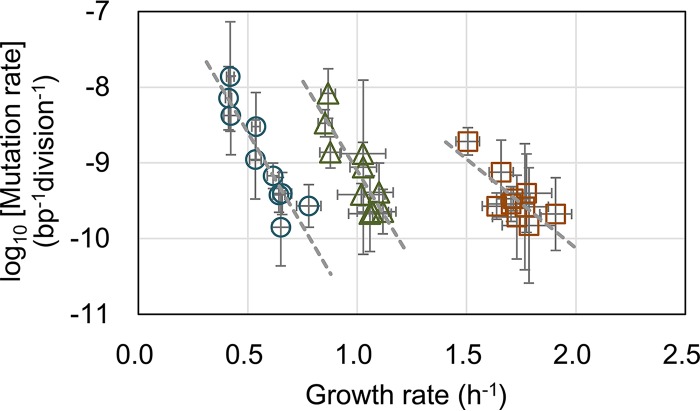
Correlations between growth rate and mutation rate in three media. The mutation rates of both the wild-type genome (no. 0) and the nine reduced genomes (nos. 3, 4, 10, 11, 14, 19, 23, 27, and 28) were plotted against their growth rates. The results in the growth media LB, MAA, and M63 are represented as brown squares, green triangles, and blue circles, respectively. Spearman rank correlation coefficients of the growth and mutation rates were −0.648 (*P* = 0.04), −0.782 (*P* = 0.008), and −0.915 (*P* = 2e−4), in LB, MAA, and M63, respectively. Standard deviations of both growth and mutation rates are indicated. Gray broken lines indicate the linear regression of the growth rates and the logarithmic mutation rates. The slopes are −4.9 (*r*^*2*^ = 0.83), −4.8 (*r*^*2*^ = 0.76), and −2.4 (*r*^*2*^ = 0.59) for M63, MAA, and LB, respectively.

Due to the similarities between the changes in the mutation and growth rates, we assumed that the genome reduction-induced changes in the mutation rate were associated with changes in the growth rate. Comparison of the growth and mutation rates of the 10 strains revealed a strong tendency of increased mutation rates with decreasing growth rates (Fig. 3). Negative correlations between the growth rate and mutation rate were commonly detected across genomic variations under all nutritional conditions (*P* < 0.05). This finding was consistent with those of previous studies that showed the evolution of decreased mutation rates accompanied by increased fitness (4) and fitness-correlated mutation rate plasticity in a single genotype (24). However, the magnitudes of the coordinated changes between the growth and mutation rates were nutritionally differentiated, which is quantitatively proposed to following this equation: log_10_(*M*_*i*_) = log_10_(*M0*) + (*α* × μ_*i*_).

Here, *M*_*i*_ and *μ*_*i*_ represent the mutation rate and the corresponding growth rate, respectively, under a certain condition. *M0* and *α* indicate the maximal mutation rate when the growth rate decreases to zero and the rate of the order decrease in mutation rate resulting from the increase in growth rate (i.e., the slope), respectively. Both *M0* and *α* are nutrition dependent but independent of the genome length. Regression of the experimental data sets showed *α* to be −4.9, −4.8, and −2.4 in M63, MAA, and LB, respectively (Fig. 3, gray lines), indicating that the growth decrease that was correlated with the increase in the mutation rate in poor media (M63 and MAA) was approximately two orders greater than that in the rich LB medium. However, the mutation capacity *M0* was approximately 4e−6 (10^−5.4^), 5e−5 (10^−4.3^), and 8e−7 (10^−6.1^) per base pair per division in cells grown in LB, MAA, and M63, respectively, which was not based on nutritional richness, suggesting that amino acids (in MAA) might play a particular role in DNA replication fidelity for reduced genomes. The results strongly indicated a quantitative relationship between the mutation and growth rates with genome reduction.

### The decrease in mutation rate is coordinated with a fitness increase in experimental evolution.

**FIG 4:**
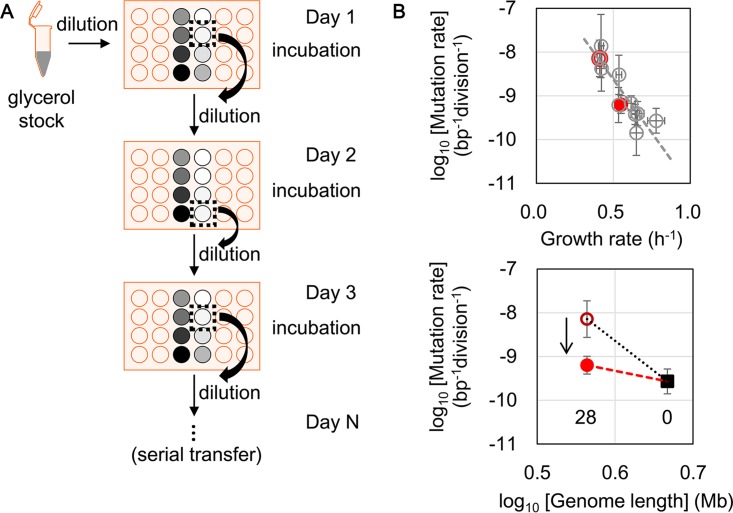
Changes in growth and mutation rates due to serial transfer**.** (A) Schematic of the serial transfer step. Reduced genome no. 28 was transferred into M63 medium in 24-well microplates (light orange). The gradations in gray circles show the variations in cell density (based on the OD_600_) of overnight culture in each well. Dotted squares indicate wells for which the OD_600_ was between 0.001 and 0.05; these wells were then selected for the dilution and incubation steps. (B) Decrease in mutation rate resulting from the serial transfer. Growth increase that accompanied the decrease in mutation rate (upper) and the declined rate of genome length-associated changes in mutation rate (bottom) are shown. The reduced genome of no. 28 is highlighted in red. Before and after the serial transfer into M63 medium are indicated as red open and filled circles, respectively. The gray open circles and the broken lines represent the same conditions as described for Fig. 3, under growth in M63. The filled square and the arrow indicate the wild-type genome no. 0 and the direction of experimental evolution (serial transfer), respectively. The dotted and broken lines roughly indicate the rates of genome size-correlated changes in mutation rate before and after the serial transfer, respectively.

To verify the correlation between the growth rate and the mutation rate, reduced genome no. 28, which retained approximately 80% of the wild-type genome sequence, was subjected to serial transfer in M63 (Fig. 4A). The cells were transferred daily during the early exponential phase for approximately 2 months, which was similar to methods used in evolution experiments that are commonly performed to increase the growth fitness of the target *E. coli* strain under defined conditions (2, 3). The evolved reduced genome no. 28, which experienced approximately 400 generations of serial transfer, acquired a 1.3-fold increase in fitness associated with approximately a 1-order decrease in mutation rate (Fig. 4B). The experimental evolution triggered an accelerated growth rate with a decreased mutation rate (Fig. 4B, highlighted in red). Consequently, the changes in mutation rate were dynamically coordinated and negatively correlated with the changes in growth rate, which was consistent with the theoretical hypothesis of predicted fitness-dependent mutation rates in mathematical simulations (25, 26) and experimental evidence of decreased growth in reduced genomes (19, 20).

Additionally, the growth rate-mediated changes in the mutation rate followed roughly the trajectory estimated in the second equation discussed above, log_10_(*M*_*i*_) = log_10_(*M0*) + (*α* × μ_*i*_) (Fig. 4B, upper panel, broken line), suggesting that this trajectory was a common path for the coordination between growth and mutation rates in M63. Moreover, the experimental evolution of a time scale of ~400 generations reduced not only the mutation rate but also the rate (*r*) of the genome size-correlated changes in mutation rate by roughly 4-fold (Fig. 4B, bottom panel). As the evolved reduced genome still retained a relatively high mutation rate, the result well explained why the magnitude of genome size-correlated changes in mutation rate was much lower during genome evolution than that observed in genome reduction, as estimated with the first equation discussed above, log_10_(*M*_*i*_) = *r*[log_10_(*G*_*i*_)] + *b.*

### Global coordination between growth and mutation rates.

**FIG 5:**
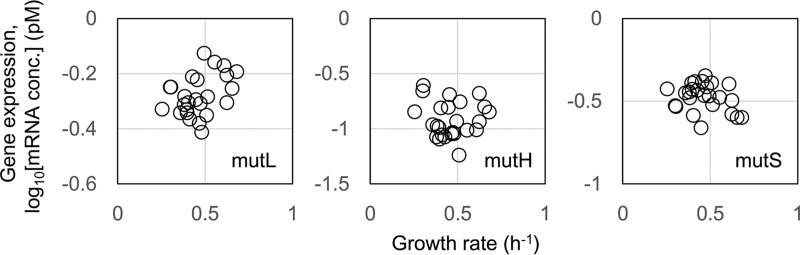
Relationships between growth rate and expression of genes responsible for mismatch repair. Expression levels of the genes that participate in mismatch repair are plotted against the growth rates. The gene *mutL*, *mutH*, and *mutS* are indicated. The correlation coefficients between the growth rates and the logarithmic gene expression levels were 0.39 (*P* = 0.05), −0.06 (*P* = 0.79), and −0.26 (*P* = 0.20) for *mutL*, *mutH*, and *mutS*, respectively.

The question of why genome reduction led to decreased fitness and increased mutation rates remains unanswered. In a view of genome evolution, genome reduction is a type of genetic interruption and is stressful to *E. coli* cells, which are accustomed to possessing a complete wild-type genome, as previously proposed (19). Stress-induced fitness decrease is a familiar phenomenon and is directly linked to transcriptome reorganization. Transcriptome reorganization is highly coordinated with growth fitness in a trade-off manner (27–30), in which the upregulation of the genes involved in stress is balanced by the downregulation of the genes that contribute to growth fitness. Accordingly, we assumed that the fitness decrease caused the transcriptional repression of the genes responsible for mismatch repair, leading to an accelerated mutation rate. To verify this assumption, microarray data sets associated with precise growth rates were collected (a total of 75 data sets), and the representative genes of *mutHLS*, of which the mutants and/or deletions showed increased mutation rates (31–33), were subjected to analysis. Unexpectedly, the expression levels of these genes showed no significant correlation with the fitness decrease (Fig. 5). The expression levels of the proteins/enzymes involved in mismatch repair were slightly changed in cells that had a decreased growth rate. The analytical results suggested that the growth rate-coordinated changes in mutation rate, which occurred in exponentially growing *E. coli* cells, were not simply due to molecular mechanisms mediated by *mutHLS* but were balanced by the global reorganization of either gene expression or cellular conditions. The previous studies found that the mismatch repair system was mostly responsible for transition mutations (34–36), which was consistent with the result that changes in the expression of the *mutHLS* system alone could lead to a moderately correlated change in the global parameters of the mutation and growth rates.

### Hypothesis regarding the comparable effects of deleted genes on growth rate.

If the reduced growth rate was the primary driver of the mutation rate, the question remained as to whether it was the reduction of genome size or the deleted genes that caused the decreased growth. As the genome reductions are always accompanied by gene deletions (11, 13, 37), direct experimental investigation would be difficult. Because the reduced genomes used in our study were constructed by removing genomic regions that did not affect *E. coli* growth or basic metabolism (13, 38), the genome reduction did not delete the genes linked to growth. Nevertheless, there is no guarantee that the deleted genes (genomic regions) did not cause a growth deficiency, because some deleted genes of unknown function or uncharacterized properties might play a role in growth fitness. To investigate the effect of gene deletions, we performed a rough survey using open-access data from the Keio Collection for single-gene knockouts in *E. coli*, which included ~3,900 strains (39). Although the data set offered the optical density (OD) values after 22 to 48 h of incubation and culture in LB or morpholinepropanesulfonic acid (MOPS) medium and did not represent the growth rates, these measurements still reflected the effects of gene deletions on growth properties. The means and standard deviations were calculated for the OD values of the knockout strain nos. 73, 112, 463, 558, 694, 816, 914, 954, and 955, for which the deleted genes were absent from the reduced genomes of nos. 3, 4, 10, 11, 14, 19, 23, 27, and 28, respectively. The results showed that the average growth and variation of the knockout strains assigned to the reduced genomes were highly equivalent (Fig. S3A). This finding indicated that the deleted genomic regions (the sum of the deleted genes without considering the gene-gene interactions) affected growth fitness to a comparable degree, partially supporting our assumption that the deleted genes all contributed slightly to growth.

In addition, the growth rates of five selected strains, which presented the lowest OD values in either LB or MOPS, were precisely evaluated in both LB and M63. There was a significant decrease in growth in strains carrying a single deletion of either JWID0986 (*yccE*) or JWID5137 (*ycdG*) (Fig. S3B). This experimental evidence demonstrated that there were several deleted genes of unclear function that affected growth fitness. However, because these two genes were both deleted in the reduced genome of strain no. 3, the growth decrease mediated by the further genome reduction (i.e., reduced genomes of nos. 4 to 28) could not be explained simply by the loss of the two genes. If the deleted genes did cause the decreased growth, then the correlation between genome length and growth rate (Fig. S2) must have been nonsignificant. Taken together, the observed significant changes in growth of the reduced genomes were due to the accumulated deletions of multiple genes. The genome reduction effect was assumed not to be simply caused by a size effect but also by the sum of the gene-gene interactions.

10.1128/mBio.00676-17.2 FIG S2  Genome length-correlated changes in growth rates**.** The growth rates of the wild-type genome W3110 (no. 0) and its reduced genomes (nos. 3, 4, 10, 11, 14, 19, 23, 27, and 28) in M63 are indicated. The Pearson correlation coefficient was 0.927 (*P* = 1e−4). Standard deviations of the repeated measurements (*n =* 12 to 24) are shown. Download FIG S2, PDF file, 0.1 MB. Copyright © 2017 Nishimura et al. 2017 Nishimura et al. This content is distributed under the terms of the Creative Commons Attribution 4.0 International license.

10.1128/mBio.00676-17.3 FIG S3  Growth was affected by single-gene deletions**.** (A) Mean growth of the single-gene knockout strains. The upper and lower panels indicate the average growth (based on the OD_600_) of the knockout strains in LB (22 h) or MOPS (48 h) medium, respectively. Genome numbers represent the reduced genomes used in the present study. The OD values of knockout genomes 73, 112, 463, 558, 694, 816, 914, 954, and 955 were analyzed for the genes absent in the reduced genomes of nos. 3, 4, 10, 11, 14, 19, 23, 27, and 28, respectively. The calculated means and standard deviations are shown. (B) The growth rates of the single-gene knockout strains. The upper and lower panels show the growth of five selected strains, in which the gene of the assigned JWID was deleted, in LB and M63 media, respectively. A growth analysis was performed as described for the genome-reduced strains. Gene numbers represent the JWID of the deleted genes. JWID0986 and JWID5137 were deleted in reduced genome no. 3; JWID1147 was deleted in no. 4; JWID1908 and JWID1557 were newly deleted in no. 11. Standard deviations of the repeated measurements (*n =* 6 to 32) are shown. Download FIG S3, PDF file, 0.1 MB. Copyright © 2017 Nishimura et al. 2017 Nishimura et al. This content is distributed under the terms of the Creative Commons Attribution 4.0 International license.

### Conclusion.

**FIG 6:**
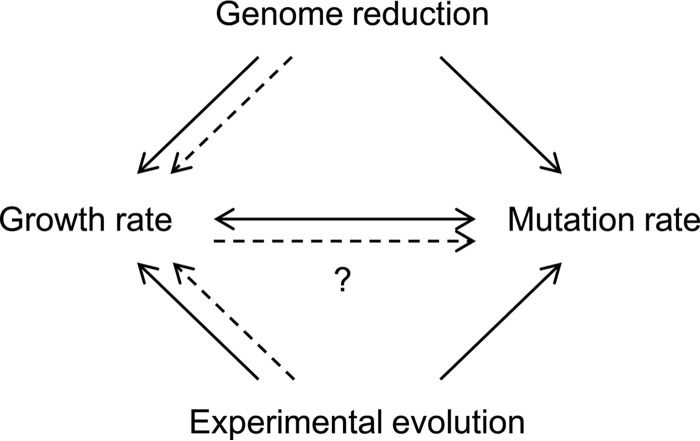
Schematic drawing as a summary. The single- and dual-directional arrows indicate the consequences of either genome reduction (Fig. 1) or experimental evolution (Fig. 4) and the coordinated relationship between growth and mutation rates (Fig. 3 and 4), respectively. The solid and broken lines represent the experimental evidence and a hypothetical mechanism of the growth rate as a driving force of the mutation rate, respectively.

The present study is the first to identify changes in mutation rates as a consequence of genome reduction and the coordinated changes in mutation and growth rates (Fig. 6). The mechanism of these correlated changes was unclear, although the growth rate was assumed to be a primary driver of mutation rates (Fig. 6). Removing nonessential genomic sequences might increase stress sensitivity, potentially accelerating genome replication errors for better growth activity. The genome length-correlated changes in the mutation rate appeared to be common in both genome evolution and genomic engineering. The increased mutation rate caused by genome reduction was largely decreased by serial transfer, reflecting the gradual changes in the mutation rate in correlation with the evolution of genome size, as occurs in nature. Genome reduction also obeyed the coordinated relationship between growth rate and mutation rate, regardless of the nutritional conditions. The correlations among the global parameters of genome size, growth rate, and mutation rate might be the fundamental working principles for maintaining cellular homeostasis.

## MATERIALS AND METHODS

### Strains and media.

Ten W3110 *E. coli* derivatives with varied genome lengths (reduced genome strain nos. 3, 4, 10, 11, 14, 19, 23, 27, and 28 and the wild-type strain 0, as previously described [19]) were selected from the KHK (Kyowa Hakko Kirin) library (13), an *E. coli* collection of reduced genomes (from the National BioResource Project, National Institute of Genetics, Shizuoka, Japan), and used in the present study. Cell culturing in three different media, complete medium (LB), minimal medium (M63), and minimal medium supplemented with 20 amino acids (MAA), was performed as previously described (19).

### Mutation rates.

The mutation rates were measured according to resistance to the antibiotic nalidixic acid, using fluctuation tests as previously reported (33). However, the number of cells was counted using a CFU assay instead of flow cytometry. Cell cultures in the exponential phase of growth were diluted 3-fold and plated onto 9 to 12 LB plates for the CFU assay. After overnight incubation, the number of colonies on each plate was determined, and only colony numbers ranging from 10 to 500 colonies per plate were considered reliable counts. The final CFU results were calculated by averaging the reliable counts of four to nine plates. More than 5,000 agar plates were used for the tests. Notably, the mutation rate was evaluated based on the emerging frequency of nalidixic acid resistance; nevertheless, we previously verified that the relative mutation rates did not change in response to different antibiotics (33).

### Growth rate.

The *E. coli* growth rate was evaluated using a 96-well microplate (Costar; Corning) with a microplate reader (Epoch2; BioTek), as described previously in detail (19). The growth rate was calculated according to the following equation: μ = [ln(*C*_*j*_/*C*_*i*_)]/(*t*_*j*_ − *t*_*i*_). A part of the growth data sets was adopted from a previous study (19).

### Serial transfer.

The serial transfer of reduced genome no. 28 was performed on 24-well microplates specific for microbe culture (Iwaki). The glycerol stock of genome no. 28 was initially inoculated in M63 and cultured until reaching the exponential phase. The cell culture was subsequently diluted 8-fold (10 to 10^8^) with fresh M63 medium in eight different wells of a new 24-well microplate. Each well contained 1.8 ml of cell culture. The plate was incubated overnight in a microplate bio-shaker (Deep Well Maximizer; Taitec) at 37°C, with rotation at 500 rpm. Only one of the eight wells (dilutions) showing growth in the early exponential phase (OD_600_ of 0.001 to 0.05) was selected and diluted into eight wells of a new plate using eight dilution ratios. The serial transfer was repeatedly performed for 50 days, equivalent to approximately 400 generations. The mutation and growth rates of the evolved genome no. 28 were measured as described above.

### Bioinformatic data sets and analyses.

Information on the deleted genomic sequences (KHK collection) was obtained from the National BioResource Project website (http://shigen.nig.ac.jp/ecoli/strain/) as previously described (19). The accumulated length of deleted genomic sequences was calculated in accordance. The numbers of deleted genes and the corresponding gene categories, as classified by Riley et al. (23), were determined and subjected to the correlation analysis, as previously described (19). The data sets used for the gene expression analysis were obtained from the NCBI Gene Expression Omnibus database under the GEO series accession numbers of GSE61749, GSE55719, and GSE52770. These data sets comprised large variations in either genotype or growth condition. A total of 75 raw data sets (microarrays) were subjected to global normalization, resulting in a common mean value (logarithmic value). The biological replicates were averaged to generate a representative gene expression value under each condition. The growth data sets of the exponentially growing *E. coli* cells were adopted from the associated papers (30, 40–42).
