# Supplementary figures and images for: Coordinated Changes in Mutation and Growth Rates Induced by Genome Reduction
Source: mBio. 2017 Jul 5;8(4):e00676-17. doi: 10.1128/mBio.00676-17 (PMC5573674; doi:10.1128/mBio.00676-17)

Fig. S1

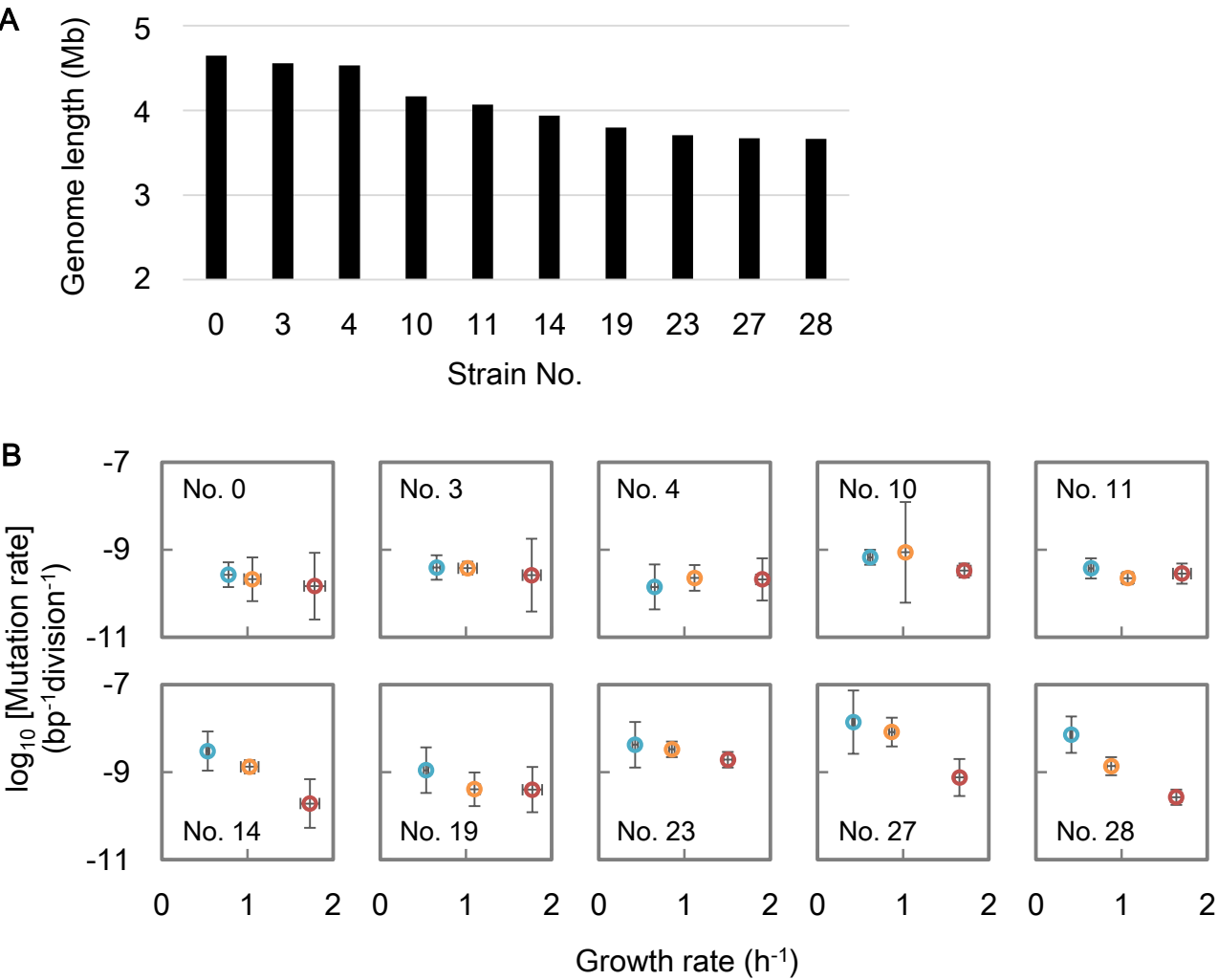

Supplement: FIG S1 [file mbo003173366sf1.pdf]

Fig. S2

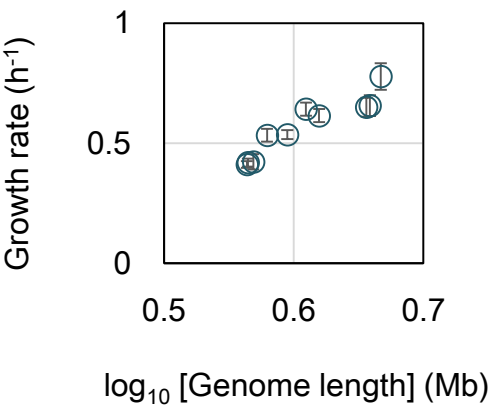

Supplement: FIG S2 [file mbo003173366sf2.pdf]

Fig. S3

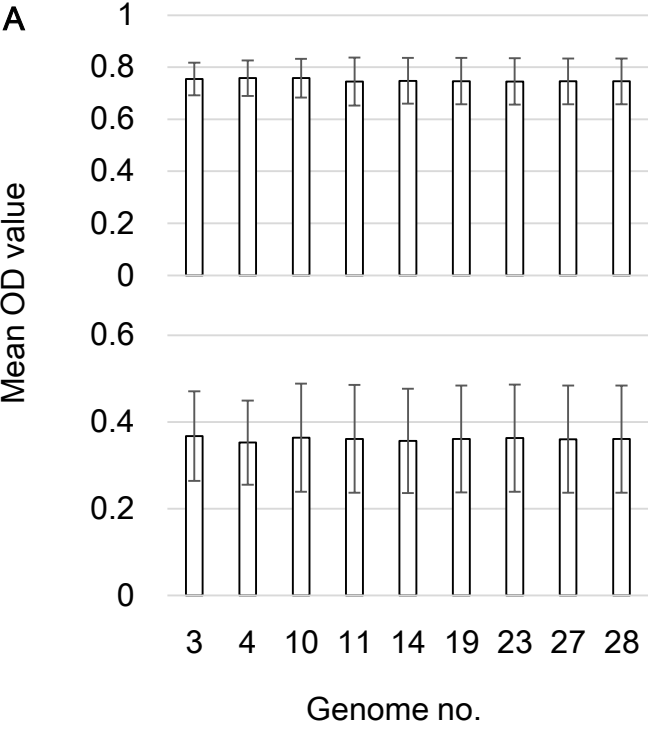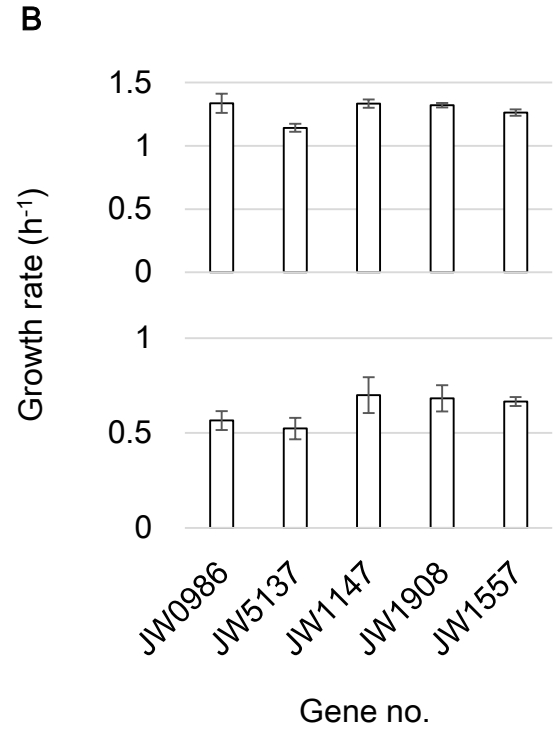

Supplement: FIG S3 [file mbo003173366sf3.pdf]
