# Supplementary material for: Coordinated Changes in Mutation and Growth Rates Induced by Genome Reduction
Source: mBio. 2017 Jul 5;8(4):e00676-17. doi: 10.1128/mBio.00676-17 (PMC5573674; doi:10.1128/mBio.00676-17)
Supplement: TABLE S1 [file mbo003173366st1.doc]

Table S1

| Strain No. | c | cp | **d** | **e** | **f** | **h** | l | lp | m | n | **o** | pc | **pe** | **pf** | ph | **pm** | **pr** | ps | **pt** | **r** | **s** | **su** | **t** | Total del. |
| --- | --- | --- | --- | --- | --- | --- | --- | --- | --- | --- | --- | --- | --- | --- | --- | --- | --- | --- | --- | --- | --- | --- | --- | --- |
| 3 | 2 | 1 | 5 | 7 | 5 | 5 | 0 | 1 | 0 | 0 | 12 | 2 | 5 | 0 | 0 | 6 | 5 | 0 | 5 | 5 | 0 | 3 | 8 | 77 |
| 4 | 2 | 1 | 5 | 7 | 5 | 38 | 0 | 1 | 0 | 0 | 24 | 2 | 7 | 0 | 0 | 7 | 7 | 0 | 6 | 5 | 0 | 5 | 8 | 130 |
| 10 | 4 | 6 | 16 | 67 | 12 | 147 | 0 | 3 | 2 | 3 | 75 | 7 | 48 | 8 | 1 | 24 | 37 | 1 | 37 | 32 | 12 | 16 | 31 | 589 |
| 11 | 4 | 6 | 19 | 74 | 13 | 173 | 0 | 3 | 2 | 4 | 83 | 8 | 51 | 8 | 1 | 26 | 38 | 1 | 38 | 34 | 12 | 19 | 32 | 649 |
| 14 | 4 | 6 | 24 | 84 | 13 | 178 | 0 | 4 | 2 | 7 | 95 | 8 | 59 | 10 | 1 | 28 | 44 | 1 | 46 | 41 | 12 | 23 | 41 | 731 |
| 19 | 4 | 7 | 26 | 105 | 16 | 213 | 0 | 4 | 2 | 7 | 120 | 8 | 72 | 10 | 4 | 38 | 49 | 1 | 58 | 47 | 17 | 29 | 50 | 887 |
| 23 | 5 | 7 | 30 | 111 | 18 | 229 | 0 | 5 | 2 | 7 | 125 | 9 | 81 | 11 | 4 | 42 | 51 | 1 | 59 | 49 | 18 | 29 | 68 | 961 |
| 27 | 7 | 7 | 33 | 115 | 18 | 229 | 0 | 6 | 2 | 7 | 128 | 9 | 85 | 11 | 4 | 44 | 51 | 1 | 75 | 50 | 18 | 29 | 69 | 998 |
| 28 | 7 | 7 | 33 | 116 | 18 | 229 | 0 | 7 | 2 | 7 | 131 | 9 | 85 | 11 | 4 | 45 | 51 | 1 | 77 | 50 | 18 | 30 | 79 | 1017 |
| All genes | 77 | 56 | 145 | 1090 | 145 | 273 | 11 | 43 | 43 | 153 | 439 | 41 | 388 | 55 | 10 | 206 | 158 | 34 | 250 | 233 | 83 | 85 | 337 |  |
